# Unraveling patient heterogeneity in complex diseases through individualized co-expression networks: a perspective

**DOI:** 10.3389/fgene.2023.1209416

**Published:** 2023-08-10

**Authors:** Verónica Latapiat, Mauricio Saez, Inti Pedroso, Alberto J. M. Martin

**Affiliations:** ^1^ Programa de Doctorado en Genómica Integrativa, Vicerrectoría de Investigación, Universidad Mayor, Santiago, Chile; ^2^ Vicerrectoría de Investigación, Universidad Mayor, Santiago, Chile; ^3^ Laboratorio de Redes Biológicas, Centro Científico y Tecnológico de Excelencia Ciencia & Vida, Fundación Ciencia & Vida, Santiago, Chile; ^4^ Centro de Oncología de Precisión, Facultad de Medicina y Ciencias de la Salud, Universidad Mayor, Santiago, Chile; ^5^ Laboratorio de Investigación en Salud de Precisión, Departamento de Procesos Diagnósticos y Evaluación, Facultad de Ciencias de la Salud, Universidad Católica de Temuco, Temuco, Chile; ^6^ Escuela de Ingeniería, Facultad de Ingeniería, Arquitectura y Diseño, Universidad San Sebastián, Santiago, Chile

**Keywords:** personalized medicine, omics, transcriptomic, co-expression, networks, diseases

## Abstract

This perspective highlights the potential of individualized networks as a novel strategy for studying complex diseases through patient stratification, enabling advancements in precision medicine. We emphasize the impact of interpatient heterogeneity resulting from genetic and environmental factors and discuss how individualized networks improve our ability to develop treatments and enhance diagnostics. Integrating system biology, combining multimodal information such as genomic and clinical data has reached a tipping point, allowing the inference of biological networks at a single-individual resolution. This approach generates a specific biological network per sample, representing the individual from which the sample originated. The availability of individualized networks enables applications in personalized medicine, such as identifying malfunctions and selecting tailored treatments. In essence, reliable, individualized networks can expedite research progress in understanding drug response variability by modeling heterogeneity among individuals and enabling the personalized selection of pharmacological targets for treatment. Therefore, developing diverse and cost-effective approaches for generating these networks is crucial for widespread application in clinical services.

## 1 Introduction

Complex diseases arise from the intricate interplay of multiple genetic and environmental risk factors. The phenomenon of simplexity, where simplicity at the phenotypic level coexists with complexity at lower organizational and molecular levels (Stewart and Cohen, 2000; Kauffman et al., 1993), suggests the existence of disease subtypes (Wallstrom et al., 2013) and emphasizes the uniqueness of each patient despite shared characteristics with others (Smith, 2011). Unfortunately, most approaches to studying complex diseases rely on identifying differences between groups based on average biomarker values, overlooking the intricate biological intricacies of these diseases. For this reason, it is necessary to use a more holistic approach that considers the molecular complexity of diseases, which involves thousands of genes across multiple cell types in different body parts (H. Zhang et al., 2019) and poses challenges for developing personalized, targeted therapies (Sierksma et al., 2020; Rouzier et al., 2005; Shipitsin et al,. 2007; Charitou et al., 2016; Khurana et al., 2013; Chan and Loscalzo, 2012).

Network biology is a rapidly developing area of research that recognizes that biological processes are not chiefly controlled by individual proteins or by discrete, unconnected linear pathways but rather by a complex system-level network of molecular interactions (X.-M. Zhang et al., 2021; Khurana et al., 2013; Charitou et al., 2016). Graph neural networks and deep-learning-based data integration models can predict disease progression and identify disease subtypes more accurately by integrating multimodal data from disparate sources, such as genetic, clinical, and imaging data (X.-M. Zhang et al., 2021; Zhou et al., 2022). Therefore, a more holistic approach that considers the molecular complexity of diseases and integrates multimodal data can provide a more comprehensive understanding of complex diseases, leading to the development of personalized, targeted therapies and improved patient outcomes in the era of precision medicine.

Cancer is a prime example of disease heterogeneity, where variability exists in various aspects, including driver mutations, making it challenging to identify causal mutations from an average view of the entire patient cohort (Lengerich et al., 2018). Moreover, diseases such as Autism spectrum disorders and epilepsy exhibit vast degrees of heterogeneity at multiple levels, including genotypes and phenotypes, resulting in diverse clinical differentiations and treatment responses (Lombardo et al., 2019). The clinical variability observed in diseases like Parkinson’s and Alzheimer’s further highlights the need to go beyond mean values and explore other approaches that capture the heterogeneous nature of complex diseases (Freudenberg-Hua et al., 2018; Ma et al., 2018).

Clinical studies of diseases often suffer from biases due to demographic, social, genetic, and ethnic factors, leading to the underrepresentation of specific population groups (Prosperi et al., 2018). This underrepresentation hampers the generalizability of conclusions to a larger population, hindering the development of effective treatments (Kessler et al., 2016; Popejoy and Fullerton, 2016; Popejoy et al., 2018; Gurdasani et al., 2019). The failure of numerous clinical trials and the lack of a cure for diseases like Alzheimer’s emphasize the need to account for population heterogeneity in trial design and consider the underlying biological mechanisms for disease subtyping (Devi and Scheltens, 2018).

While challenges exist in identifying biomarkers for heterogeneous diseases, scale-out learning approaches often need more specificity and may not be applicable in clinical practice (Khurana et al., 2013). Additionally, invasive and costly procedures or limited access to relevant tissues hinder studying central nervous system diseases (Koníčková et al., 2022). Therefore, it is necessary to adopt new approaches that precisely consider the underlying biological mechanisms in disease subtyping (Yin et al., 2019), incorporating clinical and omics analyses to improve treatment responses (Zhou et al., 2022; X.-M; Zhang et al., 2021).

The study of complex diseases is not only a scientific effort but also a public health concern. The increasing availability of drugs that can contribute to molecular-tailored treatments based on predictive biomarkers underscores the importance of improving our understanding of individual patients to enhance their quality of life (Zhou et al., 2022). To address these challenges, we require new approaches that exponentially scale up learning on complex diseases, enabling a deeper understanding of each individual and more effective interventions (X.-M. Zhang et al., 2021). By embracing these novel approaches, we can advance our knowledge of complex diseases, refine disease subtyping, and guide the selection of personalized treatment strategies to improve patient outcomes and enhance public health.

### 1.1 Individualized networks and personalized medicine

Individualized networks and personalized medicine are essential for accelerating the development of new therapies for complex diseases. Unlike the current reductionist approach, we require a system-level understanding of individuals, which can be achieved through biological networks (Ahn et al., 2006; Younesi and Hofmann-Apitius, 2013). Biological networks provide a systems-level understanding of disease mechanisms, enabling the identification of differential molecular mechanisms altered in different subtypes of disease and the disease’s progression trajectory. Networks integrate data from multiple patients to predict disease subtypes and progression, facilitating the identification of prognostic biomarkers (Furlong, 2013; Younesi and Hofmann-Apitius, 2013; McGillivray et al., 2018). Computational strategies for biological network inference have been developed to improve our understanding of biological systems (Browne et al., 2009; Liu et al., 2016; Lengerich et al., 2018; Van Der Wijst et al., 2018; Zanin et al., 2018).

Developing new therapies requires a system-level understanding of individuals with complex diseases. Biological networks are a powerful tool for this approach, enabling the modeling of complex systems (Ahn et al., 2006; Younesi and Hofmann-Apitius, 2013). By integrating data from several patients, biological networks can predict differential molecular mechanisms altered in different disease subtypes and identify the progression trajectory of the disease (Fröhlich et al., 2018). Network analysis can lead to identifying prognostic sets of biomarkers and constructing explanatory models proving their value for precision medicine. Computational strategies through biological network inference have been developed and widely validated to improve our understanding of biological systems (Browne et al., 2009). Networks can be analyzed based on graph theory tools, such as determining node properties like degree, betweenness, and other centralities (Mulder et al., 2014), and global or local graph-theoretical features describing the network may constitute potential prognostic biomarkers instead of or in addition to traditional covariates. Machine learning and artificial intelligence techniques have been employed to analyze networks (Zitnik and Leskovec, 2017; Agrawal et al., 2018; Ma et al., 2018; Zitnik et al., 2018), allowing for the identification of gene signatures that serve as prognostic markers, as demonstrated in clear renal cell carcinoma patients (Büttner et al., 2019). Several authors have developed computational strategies through biological network inference (Liu et al., 2016; Lengerich et al., 2018; Van Der Wijst et al., 2018; Zanin et al., 2018), and network-based analytics plays an increasingly important role in precision medicine (W. Zhang et al., 2017). These strategies provide a comprehensive approach to modeling biological systems, enabling construction of explanatory models that can inform precision medicine.

Furthermore, individual-specific network analysis is valuable for prediction modeling in medicine and applied health research, identifying potential prognostic biomarkers, and discovering relationships between gene modules and disease traits. Addressing these points would make the perspective more informative and engaging for readers interested in personalized medicine and the use of biological networks, machine learning, and artificial intelligence in disease research. However, it is important to carefully validate and interpret the results of the network-based analysis to ensure that they are biologically meaningful and clinically relevant (Sonawane et al., 2019; Galindez et al., 2023). Therefore, the clinical application of precision medicine will likely require a fusion of approaches tailored to each clinical problem (Duffy, 2016).

Individualized networks provide a powerful data integration and analysis paradigm, offering a systems-level understanding of disease mechanisms and underlying causes (Furlong, 2013; McGillivray et al., 2018). Combining biomedical data with appropriate network modeling approaches makes it possible to derive disease-associated information and outcomes, including biomarkers, therapeutic targets, phenotype-specific genes, survival prediction, and interactions between molecules and disease subtypes (Sonawane et al., 2019). An emergent area known as Network Medicine (Loscalzo, 2019), these approaches have allowed the stratification of cancer into subtypes predictive of clinical outcomes, such as response to therapy, patient survival, and tumor histology (Hofree et al., 2013). However, there are limitations to network-based approaches for precision medicine, such as accounting for patient heterogeneity and variability and constructing appropriate network models that depend on study design, molecular entities measured, and the type and size of data (Sonawane et al., 2019). The field should strive to integrate genomic and clinical data to build networks that detect differences for each sample. This new avenue will allow us to classify complex diseases into clinically and biologically homogeneous subtypes, leading to a better understanding of disease pathophysiology and developing more targeted interventions (Sørlie et al., 2001). By employing computational and systems biology applications to develop individualized protocols, it is possible to minimize patient suffering while maximizing treatment effectiveness, allowing for the progression of precision medicine and exploring differences between individuals (Barh et al., 2020).

The advantage of individualized protocols seen from the network paradigm over other strategies is that we can study one network per sample, make identification of modules in each network, compare patients by comparing their respective networks, cluster individuals based on sample-specific networks, and associate networks (sub-)structure to disease status (more detailed in Table 1).

**TABLE 1:** Summary of study design in biological networks.

	Networks in a whole population	Case versus control network comparison	Personalized networks
Experimental design	Generation of one network from a population	Generation of two or more networks representing cases and controls	Generation of one network per sample/individual
Analytical protocol	To obtain network modules and associate each of them with disease status	To find condition-specific clusters of individuals based on the comparison of networks	Network comparison to identify modules for each sample
		To identify structural network differences associated with modules in disease status	Association of network structure and the presence/absence of modules to disease status
Pros	Allow study correlation relation among genes in samples	Allow finding in general sense differences and making comparisons among control and case samples.	Network for each individual allows representing of what happens in each subject
cons	The resultant network does not represent the variation in the population	Network of cases and control allows represent a consensus of the group studied	Coexpression network methods have minimal samples to consider in analysis (30 samples) to reach optimal performance

### 1.2 Approaches for generating individualized networks

**FIGURE 1:**
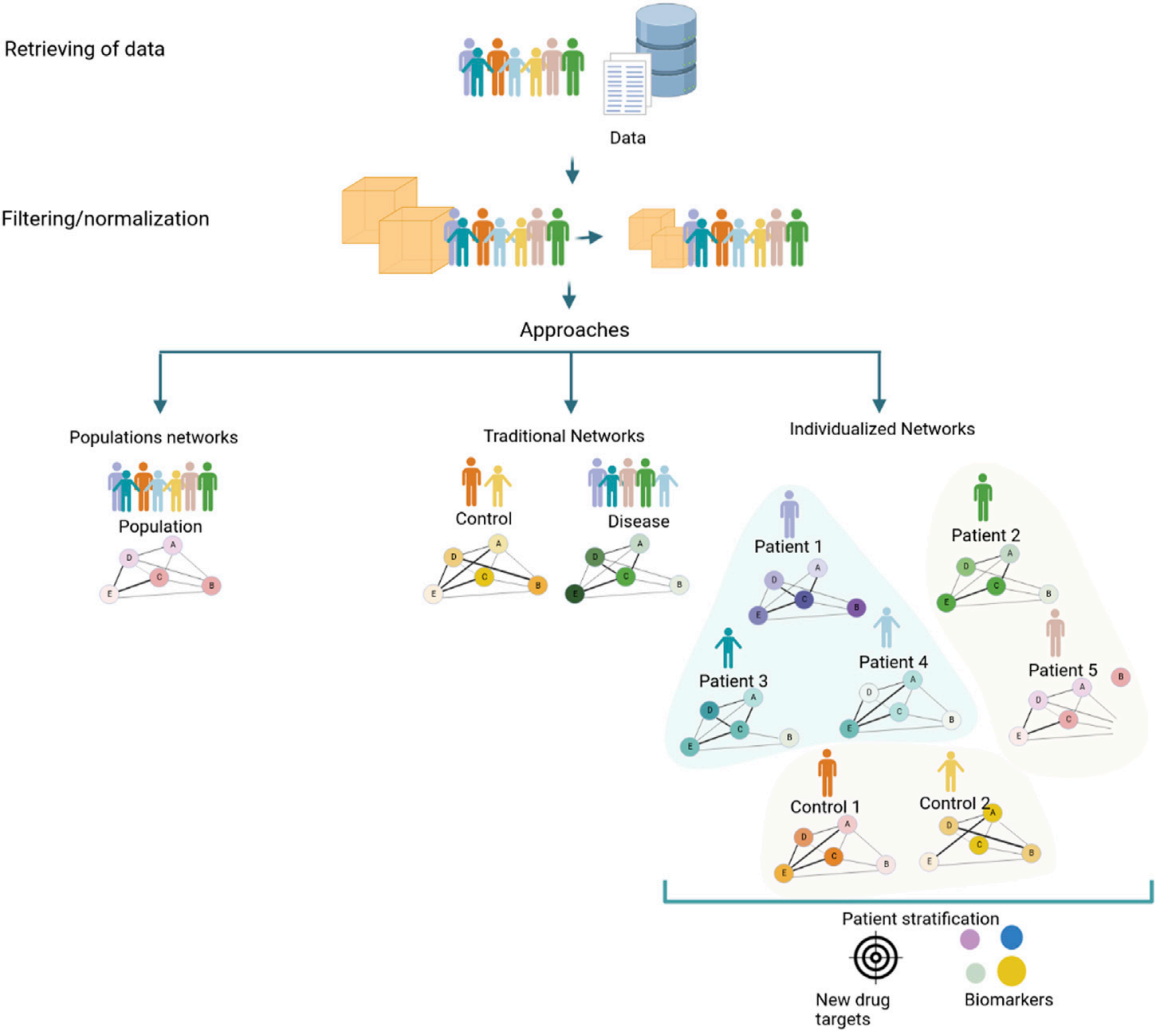
Strategies to generate a coexpression network using a conventional approach that implies a population network, a traditional (control/diseases) network, and the new individualized coexpression approach. The network generation process to generate networks with different approaches consists of a series of steps: obtention of data from patients, clinics, and/or databases, normalizing data, and filtering features for ameliorating inconsistencies. Strategies commonly employed in studies of diseases through networks, population, and traditional (case and control) networks consider mean values of populations that limit known processes that can occur in unique patients; for this reason, individualized networks between genes in samples could trigger give knowledge about changes at the level of pathways associated with diseases, with the potential to discover new drug targets and biomarkers.

Nonetheless, it is possible to identify pathways and further elucidate the molecular mechanisms of disease for individual patients using biological systems strategies. Evaluating correlations or other quantitative measures between molecules for each individual, which are usually unavailable in clinical practice, is the goal of the individualized network approach. However, this requirement for molecular data seriously limits the application of this methodology in personalized medicine (Galindez et al., 2023). Recently, several authors have developed new strategies to infer networks at the individual level, which can facilitate the discovery of differentiated disease modules or different candidate mechanisms. Although the traditional aggregated or averaged networks have allowed us to gain important insights across a wide range of biological systems and diseases, they only capture processes shared across a population of samples (Figure 1). Therefore, individualized network approaches have the potential to advance precision medicine by enabling the identification of molecular pathways that underlie complex disease phenotypes (Van Der Wijst et al., 2018; Galindez et al., 2023).

Each of the individualized networks is representative of the wiring of a specific individual and can characterize the specific disease state of an individual, as opposed to more traditional methods in which the network represents a population or cohort (Sonawane et al., 2019). Moreover, several approaches have been suggested for exploring sample-level network information (Zanin et al., 2018; Liu et al., 2016; Kuijjer et al., 2019; Dai et al., 2019; Campos-Laborie et al., 2019; X. et al., 2021) (summarized in Table 2). Furthermore, several authors focus on single-cell analysis due to the sparsity and heterogeneity of transcript counts. Authors such as (Liu et al., 2016; Liu et al., 2016; Dai et al., 2019; Dai et al., 2019) used individualized network strategies to study scRNA-seq heterogeneity in different cell types present in the same sample (R.-S. et al., 2023). These methods can also be applied similarly to construct individual networks of each bulk RNA-seq patient data sample. However, there are potential challenges and limitations in multi-omics network medicine approaches, and the clinical application of precision medicine will likely require a fusion of approaches tailored to each clinical problem (Duffy, 2016; Sonawane et al., 2019). To use knowledge of individualized biological co-expression networks in clinical settings its necessary collect individual-level data, construct and analyze co-expression networks to detect disease-relevant gene clusters and identify personalized biomarkers and therapeutic targets (Harikumar et al., 2021). This analysis can guide the selection of personalized therapies, leading to improved treatment outcomes and reduced side effects. Therefore, it is important to carefully validate and interpret the results of individualized network approaches to ensure that they are biologically meaningful and clinically relevant (Galindez et al., 2023).

**TABLE 2:** Summary of sample-specific methods.

Method	Type of network (nodes/edges)	Context
Convergence/divergence network creation Zanin, Tuñas, and Menasalvas. (2018)	Nodes correspond to the study subjects. Weight is further associated with the link between two nodes representing the distance between their features	Works assume that each disease is characterized by a high internal coherence (or homogeneity), but they explore the opposite possibility in this work
Sample specific network Zanin, Tuñas, and Menasalvas. (2018); Liu et al. (2016); Kuijjer et al. (2019); Dai et al. (2019); Campos-Laborie et al. (2019); Wang, Choi, and Roeder. (2021)	Nodes correspond to genes. Edge represents the distance between their genes	They developed a statistical method that allows constructing of individual-specific networks based on molecular expressions of a single sample to characterize various human diseases at a network level
LIONESS (Linear Interpolation to Obtain Network Estimates for Single Samples) Zanin, Tuñas, and Menasalvas. (2018); Liu et al. (2016); Kuijjer et al. (2019); Dai et al. (2019); Campos-Laborie et al. (2019); Wang, Choi, and Roeder. (2021)	Model regulatory network in individual samples. Network in which “nodes” represent genes and “edges” represent a single estimate for the likelihood of interaction between those genes	Aggregate or traditional network models fail to capture population heterogeneity. They propose a method to reverse engineer *sample-specific* networks from aggregate networks. They used these networks to study changes in network topology across time and to characterize shifts in gene regulation using linear interpolation to the predictions made by existing aggregate network inference approaches
Cell-specific network Zanin, Tuñas, and Menasalvas. (2018); Liu et al. (2016); Kuijjer et al. (2019); Dai et al. (2019); Campos-Laborie et al. (2019); Wang, Choi, and Roeder. (2021)	Nodes are genes and edges are gene–gene associations, based on statistical dependency	This method transforms the data from ‘unstable’ gene expression form to ‘stable’ gene association form on a single-cell basis to obtain a network for one cell from scRNA-seq data. This method can find differential gene associations for every single cell. Traditional differential gene expression analyses ignore even ‘dark’ genes that play important roles at the network level. And can be applied to construct an individual network of each sample bulk RNA-seq data
locCSN Zanin, Tuñas, and Menasalvas. (2018); Liu et al. (2016); Kuijjer et al. (2019); Dai et al. (2019); Campos-Laborie et al. (2019); Wang, Choi, and Roeder. (2021)	Nodes are genes, and edges are gene–gene associations	They develop an approach that estimates cell-specific networks for each cell, preserving information about cellular heterogeneity that is lost with other approaches

### 1.3 The potential of individualized gene networks in personalized medicine

Individualized gene networks have emerged as valuable tools for personalized medicine, allowing for identifying disease-associated biomarkers with diagnostic and prognostic value (Emmert-Streib et al., 2014). By unraveling molecular interactions, these networks enhance the accuracy and timeliness of disease diagnosis and facilitate the selection of more effective treatment options. Furthermore, specific network-building strategies enable the prediction of individual drug responses, minimizing exposure to ineffective drugs and reducing side effects (Van Der Wijst et al., 2018). Individualized networks also reveal novel therapeutic targets specific to each patient’s genetic and molecular profile, paving the way for precise and effective therapies (Yan et al., 2022). Integrating genetic, environmental, and lifestyle factors into personalized gene regulatory networks empowers healthcare providers to predict disease risk in susceptible individuals and implement early, personalized preventive measures (Van Der Wijst et al., 2018). Moreover, studying gene networks in individual cells enables the identification of molecular markers that predict disease progression and treatment response, enabling personalized treatment and real-time therapy monitoring (Emmert-Streib et al., 2014). These advancements in personalized medicine are crucial for understanding the genetic basis of common diseases and discovering new treatments and therapies (Ahmed et al., 2020).

Network individualization significantly impacts clinical applications, treatments, medications, and omics exams, contributing to more accurate and effective medical care in personalized medicine (Infante et al., 2020). Here are some ways individualization can improve patient care:

#### 1.3.1 Personalized treatments

Understanding a patient’s genetic and molecular characteristics enables doctors to design tailored treatments, including selecting specific medications, dosage adjustments, and identifying the most effective combination therapies (Suwinski et al., 2019).

#### 1.3.2 Safer medications and therapies

Individualization helps identify patients more likely to experience side effects or adverse reactions to certain medications. By better understanding the molecular interaction networks within individual patients, personalized therapeutic targets can be identified, leading to more effective and safer treatments (Goetz and Schork, 2018).

#### 1.3.3 Personalized omics exams

Performing omics exams, such as whole genome sequencing, gene expression profiling, and protein analysis, individually provides accurate and relevant data for guiding diagnosis, prognosis, and treatment (Mathur and Sutton, 2017; Ahmed et al., 2020; Williams et al., 2022).

#### 1.3.4 Early diagnosis of genetic diseases

Individualized medicine enables omics tests, such as genome sequencing, to identify specific genetic mutations associated with diseases, allowing for accurate and early diagnosis of genetic disorders and a better understanding of genetic predisposition (Aspinall and Hamermesh, 2007).

#### 1.3.5 Facilitating drug approval

By considering patients’ genetic and molecular characteristics, individualization can identify specific subgroups that may benefit more from certain drugs, expediting the drug approval process and providing access to more effective treatments for selected patients (FDA, 2022).

## 2 Challenges and perspectives of using individualized networks in precision medicine

The challenges of using individualized networks in precision medicine include the requirement for molecular data, which is usually unavailable in clinical practice, and the need to develop new strategies to infer networks at the individual level (Van Der Wijst et al., 2018; R.-S. et al., 2023). The clinical application of precision medicine will likely require a fusion of approaches tailored to each clinical problem, which can be complex and require significant computational resources (Duffy, 2016). Furthermore, the statistical rigor of network predictions comes from the study design and the size of the datasets, which can be a limitation (Galindez et al., 2023). Current approaches may need more samples to infer coexpression networks that accurately capture the complexity of individualized networks. The search space of possible coexpression networks is vast and decreased uncertainty and reduced statistical power due to the small sample size may limit the generalizability of the constructed networks (Liesecke et al., 2019).

Obtaining many samples with comprehensive genomic data can be challenging, especially for rare diseases or specific patient populations. With limited sample sizes, the statistical power to detect meaningful coexpression relationships may be reduced, leading to false positives or missing important connections. One approach to address these limitations is leveraging existing knowledge from larger datasets or databases, incorporating prior knowledge about gene-gene interactions, regulatory relationships, or functional annotations. Integrating multi-omics data from different modalities (e.g., genomics, transcriptomics, proteomics) could provide a more comprehensive view of individual-specific networks. Collaboration among researchers and data sharing can help increase sample sizes and improve the statistical power of coexpression network inference (Escorcia-Rodríguez et al., 2023). The development of novel statistical methods specifically designed for analyzing individualized coexpression networks can improve the accuracy and reliability of the inferred networks (Yu et al., 2018).

Finally, developing more sophisticated algorithms and computational methods can help extract meaningful information from smaller sample sizes and incorporate prior knowledge, improving the accuracy and robustness of individualized coexpression networks (Colby et al., 2018). For example, Liesecke et al. proposed the idea of conserved coexpression links between two genes over several datasets, reinforcing the coexpression relationship (Liesecke et al., 2019). However, there are still challenges to overcome. When merging expression data, the size increase should outweigh the noise inclusion, and graph structure should be considered when integrating the inferences (Escorcia-Rodríguez et al., 2023). The potential bias introduced by relying on external datasets should also be considered, as they may only partially represent the specific biological context of the individual sample. Moreover, methods inferring coexpression networks should no longer be assessed solely based on standard performance metrics and graph structural properties.

Overall, while individualized networks have the potential to advance precision medicine, they require careful validation and interpretation of results to ensure they are biologically meaningful and clinically relevant. For other hand, the cost of using transcriptomic data has decreased over time, making it more accessible for researchers and clinicians, and it is important to consider the potential benefits of, and funding opportunities for research in personalized medicine; for this reason, it is addressing these challenges and limitations is crucial for their success and from a perspective. Stratification makes possible the design of new clinical trials to reevaluate previously tested drugs without such stratification and determine possible new therapies or treatments for each molecular subtype of patients (Rajewsky et al., 2020).

## 3 Conclusion

Personalized medicine, with its focus on individualized medical treatment based on patient characteristics, has the potential to revolutionize healthcare by improving patient outcomes and enhancing the quality of care. Developing individualized therapy protocols considering patient heterogeneity can minimize patient suffering while maximizing treatment effectiveness; this necessitates the refinement of disease categorization to understand the biological differences among subtypes better and guide personalized treatment strategies.

Novel individualized gene coexpression networks offer a paradigm shift in studying complex diseases by revealing patient-specific gene expression patterns and modules. By integrating multimodal information and considering patient-specific characteristics, these networks enhance our understanding of disease pathogenesis, treatment response, and diagnostic accuracy. They provide a more comprehensive understanding of complex diseases, refine disease subtyping, and guide the selection of personalized treatment strategies to improve patient outcomes.

Network medicine, which integrates diverse biological networks, is emerging as a powerful approach to offer a systems-level understanding of disease mechanisms and underlying causes. By analyzing gene-gene interactions in individual samples and systematically comparing them, we can identify pathways, subtypes of disease states, and key components in the networks that can be targeted in clinical practice. Multiscale mathematical and computational tools and integrating genomic and clinical data enable the construction of individualized networks with single-individual resolution.

While the potential impact of individualized coexpression networks on clinical practice is significant, further research and interdisciplinary collaboration are needed to realize their transformative powerfully. Standardization and robustness of data-gathering approaches, including imaging, multi-omic approaches, and clinical information, are critical for scalability to larger patient cohorts. Deep-learning-based data integration models hold promise in accurately predicting disease progression and identifying disease subtypes by leveraging multimodal data from various sources.

Addressing the limitations of current approaches to infer coexpression networks requires leveraging existing knowledge, integrating multi-omics data, collaborative efforts among researchers, and developing novel statistical methods and improved algorithms. These potential solutions represent promising directions for overcoming current limitations and advancing the inference of individualized coexpression networks.

In conclusion, individualized coexpression networks have the potential to significantly advance our knowledge of complex diseases, refine disease subtyping, and guide the selection of personalized treatment strategies. By integrating diverse biological networks and considering patient-specific characteristics, these networks enhance our understanding of disease mechanisms and improve patient outcomes in the era of precision medicine. As we continue to explore the transformative potential of network medicine, interdisciplinary collaboration, further research, and methodological advancements are vital to fully harness the power of individualized coexpression networks and improve healthcare outcomes for patients.

## Data Availability

The original contributions presented in the study are included in the article/Supplementary material, further inquiries can be directed to the corresponding authors.
