# Corrigendum: Unraveling patient heterogeneity in complex diseases through individualized co-expression networks: a perspective

**DOI:** 10.3389/fgene.2023.1286081

**Published:** 2023-09-21

**Authors:** Verόnica Latapiat, Mauricio Saez, Inti Pedroso, Alberto J. M. Martin

**Affiliations:** ^1^ Programa de Doctorado en Genómica Integrativa, Vicerrectoría de Investigación, Universidad Mayor, Santiago, Chile; ^2^ Vicerrectoría de Investigación, Universidad Mayor, Santiago, Chile; ^3^ Laboratorio de Redes Biológicas, Centro Científico y Tecnológico de Excelencia Ciencia & Vida, Fundación Ciencia & Vida, Santiago, Chile; ^4^ Centro de Oncología de Precisión, Facultad de Medicina y Ciencias de la Salud, Universidad Mayor, Santiago, Chile; ^5^ Laboratorio de Investigación en Salud de Precisión, Departamento de Procesos Diagnósticos y Evaluación, Facultad de Ciencias de la Salud, Universidad Católica de Temuco, Temuco, Chile; ^6^ Escuela de Ingeniería, Facultad de Ingeniería, Arquitectura y Diseño, Universidad San Sebastián, Santiago, Chile

**Keywords:** personalized medicine, omics, transcriptomic, co-expression, networks, diseases

In the published article, there was an error in **Affiliation** 3. Instead of “Laboratorio de Redes Biológicas, Centro Científicoy Tecnológico de Excelencia Ciencia & Vida, Fundación Ciencia & Vida, Escuela de Ingeniería, Facultad de Ingeniería, Arquitecturay Diseño, Universidad San Sebastián, Santiago, Chile”, it should be “Laboratorio de Redes Biológicas, Centro Científico y Tecnológico de Excelencia Ciencia & Vida, Fundación Ciencia & Vida, Santiago, Chile.”

In the published article, there was an error regarding the **Affiliation** for Alberto J. M. Martin. As well as having affiliation(s) 3, he should also have “Escuela de Ingeniería, Facultad de Ingeniería, Arquitectura y Diseño, Universidad San Sebastián, Santiago, Chile.”

In the published article, there was an error in the **Acknowledgements** statement. We unfortunately missing to acknowledge the USS funds that partially covered the article APC, thus this statement is incomplete “VL gratefully acknowledges ANID, Chile, for Ph.D. fellowship 21181311. PoweredNLHPC (ECM-02): this research was partially supported by the supercomputing infrastructure of the NLHPC (ECM-02). Figure was created with BioRender.com.” The correct Funding statement appears below.

“VL gratefully acknowledges ANID, Chile, for Ph.D. fellowship 21181311. PoweredNLHPC (ECM-02): this research was partially supported by the supercomputing infrastructure of the NLHPC (ECM-02). Figures were created with BioRender.com. This publication had the support of the Vicerrectoría de Investigación y Doctorados of Universidad San Sebastián–Fondo VRID_APC23/11.”

The authors apologize for these errors and state that this does not change the scientific conclusions of the article in any way. The original article has been updated.

